# Diagnostic accuracy of MRA and MRI for the bursal-sided partial-thickness rotator cuff tears: a meta-analysis

**DOI:** 10.1186/s13018-019-1460-y

**Published:** 2019-12-12

**Authors:** Tao Huang, Jian Liu, Yupeng Ma, Dongsheng Zhou, Liang Chen, Fanxiao Liu

**Affiliations:** 1 Department of Orthopaedics, Yantai Shan Hospital, Yantai, Shandong Province China; 2 Department of Orthopaedics, Zhangqiu District People’s Hospital of Jinan City, Zhangqiu District, Jinan City, Shandong Province China; 3 Department of Orthopaedics, Shandong Provincial Hospital affiliated to Shandong University, No.324, Road Jing Wu Wei Qi, Jinan, 250021 Shandong China; 4 Department of Orthopaedics, Shandong Provincial Western Hospital, No. 4, Road Duan Xing Xi, Jinan, 250022 Shandong China

**Keywords:** Rotator cuff tear, MRA, MRI, Partial-thickness, Shoulder pain

## Abstract

**Background:**

Numerous quantitatively studies have focused on the diagnosis of bursal-sided partial-thickness rotator cuff tears (RCTs); however, the accuracy of magnetic resonance imaging (MRI) and MR arthrography (MRA) remains inconclusive. This study was performed systematically to compare the diagnostic value of MRA and MRI for the bursal-sided partial-thickness RCTs.

**Methods:**

Three electronic databases, PubMed, Embase, and Cochrane Library, were utilized to retrieve articles comparing the diagnostic value of MRA and MRI for detecting bursal-sided partial-thickness RCTs. After screening and diluting out the articles that met the inclusion criteria to be used for statistical analysis, the pooled evaluation indexes include sensitivity, specificity, positive and negative predictive values, diagnostic odds ratio (DOR), and the area under the receiver operating characteristic curve (AUC).

**Results:**

Twelve studies involving 1740 patients and 1741 shoulders were identified. The pooled sensitivity, specificity, DOR, and AUC of MRA to diagnose bursal-sided partial-thickness RCTs were 0.77 (95% CI, 0.67–0.85), 0.98 (95% CI, 0.95–0.99), 73.01 (95% CI, 35.01–152.26), and 0.88 (95% CI, 0.85–0.91), respectively. The pooled sensitivity, specificity, DOR, and AUC of MRI were 0.77 (95% CI, 0.66–0.86) and 0.96 (95% CI, 0.81–0.99), and 37.12 (95% CI, 8.08–170.64) and 0.82 (95% CI, 0.78–0.85), respectively.

**Conclusions:**

This meta-analysis reveals that MRA and MRI have similar diagnostic value for the diagnosis of bursal-sided partial-thickness rotator cuff tears.

## Introduction

Tear of a rotator cuff is a common source of shoulder pain and disability. Rotator cuff tears (RCTs) may be full- or partial-thickness, with partial-thickness RCTs further divided into bursal-sided, articular-sided, and intratendinous tears. With an aging population, it is expected that the prevalence and severity of RCTs occurrence will increase [1, 2]. The formation of partial-thickness RCTs is attributable to either subacromial impingement– or intrinsic age–related phenomena [3–5]. Moreover, differential strain in the different tendon layers induces intratendinous delamination [6], which may cause various patterns such as full- and partial-thickness RCTs. Compared with interstitial- and articular-sided partial-thickness RCTs, bursal-sided tears can result in more severe shoulder pain [7], possibly because of their association with subacromial impingement [4, 8].

In the last decade, shoulder plain film and various physical examination tests have been shown to be insufficient at effectively diagnosing rotator cuff tears [9, 10]. In fact, numerous studies have reported that MRA is far more diagnostically effective for detecting full- and partial-thickness RCTs but especially small full-thickness tears [11, 12]. A previous meta-analysis [13] that utilized 65 articles suggested that MRA could provide the accuracy in detecting full-thickness tears. A recent meta-analysis [14] suggested that 3-dimensional shoulder ultrasound is very effective and highly accurate to detect full-thickness RCTs, but might lack accuracy in the diagnosis of partial-thickness tears. With the improvements in arthroscopic diagnosis and imaging modalities involving MRI, MRA, and ultrasound, the number of patients with partial-thickness RCTs diagnosed has been increasing, which enhanced the knowledge and understanding of partial-thickness RCTs. Despite improvements in detection, partial-thickness tears are less likely to heal spontaneously and they tend to progress over time [15]. However, fewer studies have focused on partial-thickness RCTs than on full-thickness RCTs, not to mention the bursal-sided tears.

Bursal-sided partial-thickness RCTs have been historically difficult to treat due to the lack of agreement on accepted treatment algorithms. Previous study [16] in our laboratory confirmed that stem cell could improve the rehabilitation of the rotator cuff disorders. Although non-surgical treatment is an effective method to reduce local inflammation in all stages of rotator cuff tendon pathology [17], a satisfactory clinical outcome from non-surgical strategy is successful in < 50% thickness tendon tears [18]. Recently, the indication for surgical treatment of partial-thickness RCTs has not been clearly established; the favorable clinical results when repairing a 50% thickness tendon tear demonstrated that 50% thickness tendon tear could be considered as a threshold for the treatment of bursal-sided partial-thickness RCTs [19–24]. For tears < 50%, conservative treatment consisting of subacromial decompression without arthroscopic repair yielded satisfactory results at a minimum 1-year follow-up [23]. A biomechanical analysis evaluating the relationship between depth of bursal-sided partial-thickness tears and strain may elucidate the underlying mechanisms behind the outcomes observed in clinical practice when using 50% thickness tears as a threshold for repair [25].

Recently, technological advances in arthroscopic shoulder surgery have made surgical management of partial-thickness tears much less invasive and thereby more cost effective. Bursal-sided partial-thickness RCTs are known to show satisfactory clinical outcomes after arthroscopic rotator cuff repair and acromioplasty [26–28]. Therefore, the identification of subtype of partial-thickness tears has become more meaningful [18, 29]. Multiple studies focused on the assessment of diagnostic value of MRA, MRI, or ultrasound imaging for full- or partial-thickness tears. However, few studies have so far been conducted to rigorously evaluate the diagnostic value of MRA and MRI for the bursal-sided partial-thickness RCTs.

As such, the present study aims to evaluate all available scientific published material to compare the diagnostic accuracy of MRA and MRI for detecting bursal-sided partial-thickness rotator cuff tears.

## Materials and methods

This meta-analysis was conducted in accordance with the Preferred Reporting Items for a Systematic Review and Meta-analysis of Diagnostic Test Accuracy Studies (PRISMA-DTA) statement [30].

### Search strategy

A comprehensive literature search of three electronic databases, including PubMed, Embase, and the Cochrane Library, was performed for entries recorded from the time of database inception to January 1, 2019. The vocabulary and syntax were specifically adapted according to the database. We used “diagnostic,” “diagnostic imaging,” “diagnosis,” “diagnostic test,” “rotator cuff,” “supraspinatus,” “infraspinatus,” “subscapularis,” “labrum,” “shoulder joint,” “subacromial impingement,” “tendenopathy,” “shoulder,” “shoulder pain,” “shoulder impingement syndrome,” or “bursitis” as our diagnosis of interest and “MRI,” “magnetic resonance imaging,” “MRA,” or “magnetic resonance arthrography” as the index tests. No language limitation or other search filters were applied. The reference lists of relevant articles and included studies were also hand searched for supplementary eligible records. Searching for studies was performed by two independent investigators independently. Any disagreement was settled through the discussion of researchers until a consensus was reached.

### Inclusion and exclusion criteria

Studies eligible for this meta-analysis needed to match all the following criteria: (1) study design, diagnostic accuracy study; (2) population, patients with a suspected rotator cuff tear; (3) MRI or MRA was performed; (4) the final diagnosis of bursal-sided rotator cuff tears was confirmed by predesigned reference standards; and (5) adequate data, including true positive (TP), false positive (FP), false negative (FN), and true negative (TN), could be extracted to construct a two-by-two contingency table to determine the diagnostic performance of index tests.

Exclusion criteria were (1) animal studies or cadaver experiments, (2) studies in which bursal-sided rotator cuff tear could not be differentiated, and (3) commentaries, letters, case-reports, reviews, or congress proceedings. The titles and abstracts were independently screened and assessed in an unblinded standardized manner for eligibility. The final decision regarding inclusion was based on the full article.

### Data extraction

The following information was extracted from each study: the first author’s surname, publication year, country of origin, participant characteristics (number, age, and gender), study design, reference standard, time from diagnostic test to reference standard, blinding, number of readers, readers’ experience, clinical findings of the shoulder, technical parameters of MRA or MRI (the administration of contrast agent [intravenous: indirect or intra-articular: direct], vendor, model, magnetic strength, method, sequence, slice thickness, analyzed image plane), and diagnostic data (number of false-/true-positive [FP/TP] and false-/true-negative [FN/TN] cases). For the diagnostic modalities, the TP, FP, TN, and FN results were derived from a two-by-two contingency table.

### Quality assessment

The methodological quality of the included studies was appraised according to the Quality Assessment of Diagnostic Accuracy Studies-2 (QUADAS-2) tool [31], which comprised of four key domains (patient selection, index test, reference standard, and flow and timing) with 11 items. The risk of bias was assessed in each domain, and concerns about the applicability were assessed in the first three domains with signaling questions. These questions were answered with “yes” for a low risk of bias/concerns, “no” for a high risk of bias/concerns, or “unclear” when relevant information was not clearly provided.

### Statistical analysis

The whole process of searching, filtering, data extraction, and quality assessment was implemented by two researchers (LFX and HT) independently and repeatedly. For any discrepancy, a consensus was reached by discussion with an arbitrator (LC). Meta-analysis was performed to assess the accuracy of MRA/MRI by calculating the pooled sensitivity, specificity, positive likelihood ratio (PLR), negative likelihood ratio (NLR), diagnostic odds ratio (DOR), and area under the receiver operating characteristic curve (AUC) utilizing the original diagnostic data after the threshold effect was tested by calculating the logarithm of sensitivity and logarithm of 1-specificity. Heterogeneity among the included studies was assessed using the *I*^2^ statistic. An *I*^2^ value of 0% implied no observed heterogeneity, and values > 40% indicated substantial heterogeneity. The meta-regression and subgroup analyses were conducted to explore the available source of heterogeneity. Publication bias was performed using Deeks’ Funnel Plot Asymmetry Test. Additionally, for the non-threshold effect, we performed meta-regression analysis and the patient sample size (≥ 100 or < 100), publication year (before or after 2014), magnetic field strength (3.0-T or not), number of readers, blinding (≥ 2 or 1), and QUADAS-2 score (≥ 10 or < 10) as well as muscle tendon were used as covariates. All meta-analyses were performed using STATA (V. 12.0, StataCorp, College Station, TX).

## Results

### Studies retrieved and characteristics

**Fig. 1:**
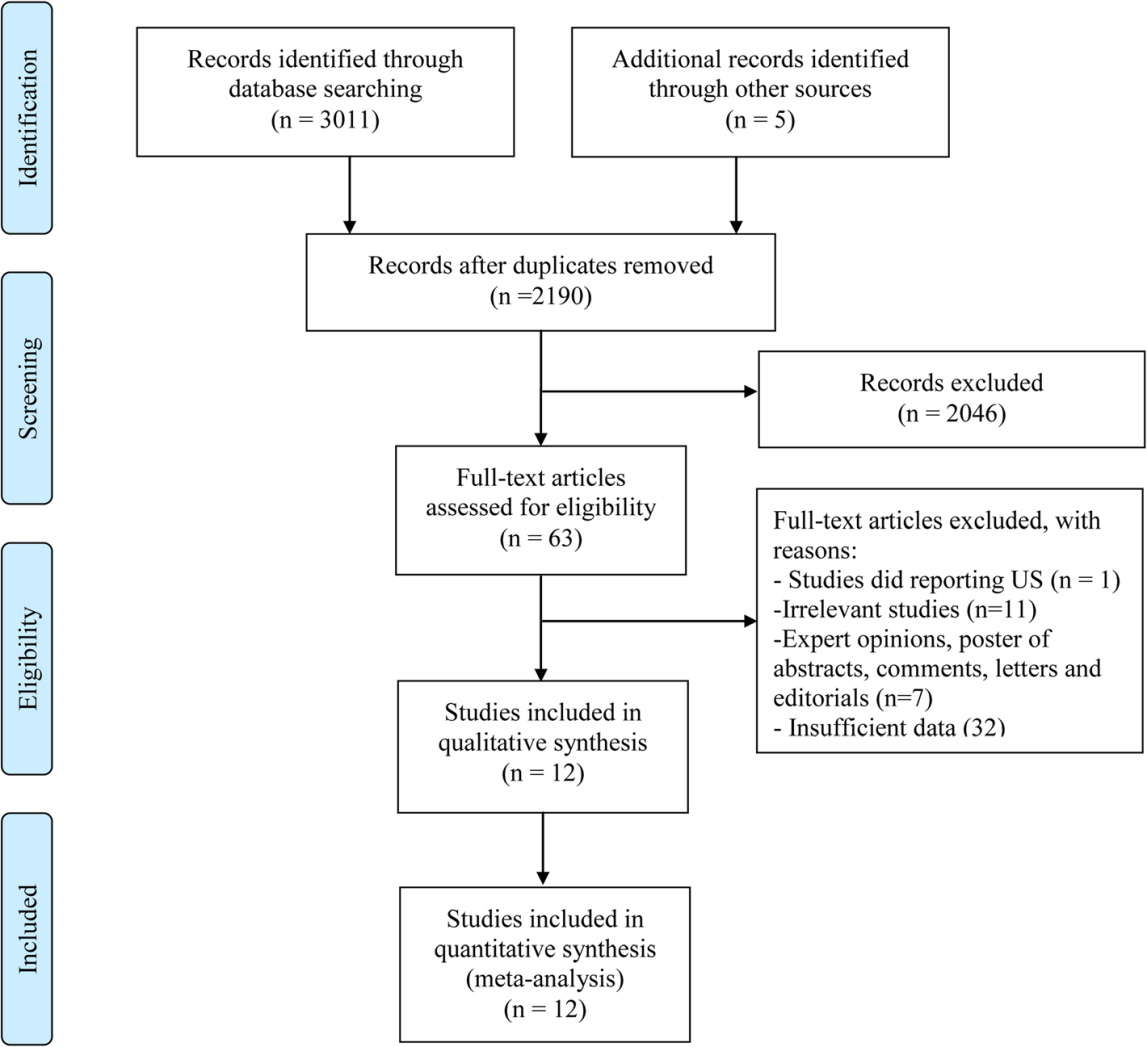
Selection process of the included studies

In total, 2190 records were identified by searching three databases and removing duplicates. The screening of the reference lists of these and other relevant articles yielded 5 additional studies. After screening remaining titles and abstracts, and identifying related full-text articles, eventually, 12 articles [29, 32–42] published during the period 2007 to 2018 remained for quantitative analysis. The selection processes for the eligible studies are summarized in Fig. 1.

The sample size of the included studies ranged from 10 to 333 with a total of 1740 patients with bursal-sided partial-thickness rotator cuff tears. Eleven studies [29, 32, 33, 35–42] used a retrospective study design, whereas the other [34] was a prospective one. Seven studies [29, 33, 37, 38, 40–42] claimed that the blinding method was used to assess the imaging modalities, while 2 studies [34, 35] did not clarify this fact. Among the included studies, 2 studies [37, 38] addressed the value of diagnostic test were evaluated by only 1 reader, 7 [29, 32–35, 40, 41] by 2 readers, and 1 [36] by 3 readers. Among the included studies, 11 studies [29, 33–42] claimed that shoulder arthroscopy was used as the gold standard. Basic information of the subjects from included studies is presented in Table 1. Main characteristics of the included studies are shown in Table 2. Main technical parameters of MRA and MRI are presented in Table 3. According to the QUADAS-2 score, 4 (33.3%) [33, 37, 41, 42], 2 (16.7%) [29, 40], 4 (33.3%) [34–36, 39], 1 (8.3%) [32], and 1 (8.3%) [38] studies scored 11, 10, 9, 8, and 7, respectively (Table 1). Additional file 1: Table S1 shows the accuracy of MRA and MRI for the detection of bursal-sided partial-thickness RCTs.

**Table 1:** Basic information of the subjects from included studies

Study	No. of patients	No. of shoulders	Mean age (age, range)	Gender (M/F)	Clinical indication of shoulder	Methods	Final diagnosis of included patients	Muscle tendon	QUADAS-2
Fritz et al. (2007)	238	238	43 (18–79)	150/80	Underwent arthroscopic or open surgical evaluation	MRI	Bursal-sided PT	SSP-ISP	8
Magee et al. (2009)	150	150	31 (14–50)	109/49	Shoulder pain	MRA/MRI	Bursal-sided PT	SSP	11
Oh et al. (2009)	36	36	53.9 (20–77)	16/20	Scheduled for shoulder arthroscopic surgery	MRA	Bursal-sided PT	SSP-ISP/SSC	9
Chun et al. (2010)	202	202	51	110/92	Shoulder pain over 3 years	MRA	Bursal-sided PT	All	10
Choo et al. (2012)	49	49	55.6 (19–71)	22/27	Shoulder discomfort	MRA	Bursal-sided PT	All	9
Modi et al. (2013)	103	103	30 (15–79)	76/27	Shoulder pain or instability symptom	MRA	Bursal-sided PT	All	9
Lee et al. (2014)	205	206	56.5 (1–78)	98/107	Undergone indirect shoulder MRA followed by arthroscopic surgery	MRA	Bursal-sided PT	SSC/SSP-ISP	11
Choo et al. (2015)	231	231	59 (21–81)	97/134	Shoulder discomfort	MRA	Bursal-sided PT	SSP-ISP	7
Farshad-Amacker et al. (2015)	37	37	47 (26–60)	26/11	Symptomatic shoulder	MRI	Bursal-sided PT	SSP	9
Lee et al. (2015)	333	333	56.9 (17–80)	160/173	Suspected of having rotator cuff lesion	MRA/MRI	Bursal-sided PT	SSP-ISP/SSC	10
Lo et al. (2016)	146	146	48.3 (19–86)	95/51	NR	MRI	Bursal-sided PT	All	11
Perez et al. (2018)	10	10	16.7 (14–18)	10/0	Shoulder pathology	MRI	Bursal-sided PT	All	11

*MRA* magnetic resonance angiography, *MRI* magnetic resonance imaging, *SSP* supraspinatus, *SSC* subscapularis, *ISP* infraspinatus, *PT* partial-thickness rotator cuff tear, *M* male, *F* female, *NR* not reported

**Table 2:** Main characteristics of the included studies

Study	Country	Inclusion interval	Study design	Gold standard	Time from MRI/MRA to gold standard, mean days (range)	Blinding	No. of readers	Reader experience (years)
Fritz et al. (2007)	USA	04.2000–07.2004	R	SA or surgery	98 (89–108)	NR	2	NR
Magee et al. (2009)	USA	01.2007–07.2007	R	SA	11 (1–30)	Yes	2	10/10
Oh et al. (2009)	Korea	03.2006–06.2006	P	SA	1	No	2	NR
Chun et al. (2010)	Korea	NR	R	SA	180 (20–150)	Yes	2	> 10
Choo et al. (2012)	Korea	08.2010–04.2011	R	SA	NR	No	2	Yes
Modi et al. (2013)	UK	11.2006–07.2011	R	SA	NR	NR	3	Yes
Lee et al. (2014)	Korea	03.2011–07.2012	R	SA	< 5	Yes	1	8
Choo (2015)	Korea	01.2011–12.2013	R	SA	7.5 (0–70)	Yes	1	8
Farshad-Amacker et al. (2015)	Switzerland	2002–2010	R	SA	NR	NR	NR	NR
Lee et al. (2015)	Korea	03.2011–09.2013	R	SA	NR	Yes	2	10/7
Lo et al. (2016)	China	01.2012–07.2013	R	SA	NR	Yes	2	20/5
Perez et al. (2018)	USA	01.2010–10.2016	R	SA	NR	Yes	NR	NR

*P* prospective, *R* retrospective, *MRA* magnetic resonance angiography, *MRI* magnetic resonance imaging, *NR* not reported, *SA* shoulder arthroscopy

**Table 3:** Main technical parameters of MRA and MRI

Study	Technical parameters							Methods
	Vendor	Model	Magnetic strength	Indirect or direct	Sequence	Slice thickness	Analyzed image plane	
Fritz et al. (2007)	GE/Siemens	NR	1.5 T	–	FS, T1WI, GE	3/4 mm	Transverse, cor obl, sag obl	MRI
Magee et al. (2009)	GE	Signa	3.0 T	NR	T1WI FSE, fs T2WI FSE	4 mm	Ax, cor obl, sag	MRA
	GE	Signa	3.0 T	–	T1WI FSE, fs T2WI FSE, fs T2WI FSE	4 mm	Ax, sag, cor obl	MRI
Oh et al. (2009)	Philips	Gyroscan Intera Achieva	3.0 T	Indirect	FS T1WI FSE, T2WI FSE, 3D fast FS GRE	3/4 mm	Ax, cor obl, sag obl	MRA
Chun et al. (2010)	Siemens	Avanto	1.5 T	NR	FS T1WI SE,T2WI FSE	3 mm	Transverse, sag obl, cor obl	MRA
Choo et al. (2012)	Philips	Achieva 3.0 T TX	3.0 T	Direct	FS T1WI FSE, T2WI FSE, 3D FS T1WI FSE	0.5/1 mm	Ax, cor obl, sag obl	MRA
Modi et al. (2013)	GE	Discovery MR750	3.0 T	NR	fs T1WI SE, STIR, fs T2WI FSE, fs T1WI SE	NR	Ax, cor obl, sag obl	MRA
Lee et al. (2014)	Philips	Gyroscan Intera Achieva	3.0 T	Indirect	FS T1WI FSE, 3D FS T1WI FSE	3 mm	Ax, cor obl, sag obl	MRA
Choo et al. (2015)	Philips	Achieva 3 T TX	3.0 T	Indirect	T2WI FSE, FS T1WI FSE	3 mm	cor obl, sag obl	MRA
Farshad- Amacker et al. (2015)	Siemens	Symphony/Espree/Avanto	1.5/3 T	–	FS PDW TSE, T1WI SE, FS T2WI TSE	3/4 mm	Ax, cor obl, sag obl	MRI
Lee et al. (2015)	Philips	Gyroscan Intera Achieva	3.0 T	Indirect	fs T1WI, T2W FSE	NR	Ax, cor obl, sag obl	MRA
	Philips	Achieva	3.0 T	–	GE, fs FSE PDW, T2WI FSE	NR	Ax, cor obl, sag obl	MRI
Lo et al. (2016)	Philips	Achieva	1.5 T	–	T1WI, FS T2WI, SE DWI	3/4 mm	cor obl	MRI
Perez et al. (2018)	NR	NR	1.5 T	–	FS T2, PD, T1WI	NR	Ax, cor obl, sag obl	MRI

*PD* proton-density, *PDW* proton-density-weighted, *FSE* fast spin-echo, *STIR* short Tau-inversion recovery, *GE* gradient echo, *TSE* turbo spin-echo, *FS* fat suppreesed, *SE* spin-echo, *GRE* gradient-recalled echo, ***sag obl*** sagittal oblique, ***cor obl*** coronal oblique, ***cor*** coronal, ***sag*** sagittal, ***Ax*** Axial, ***fs*** fat-saturated, *DE* dual-echo, *NR* not reported

### Diagnostic value of MRA

**Fig. 2:**
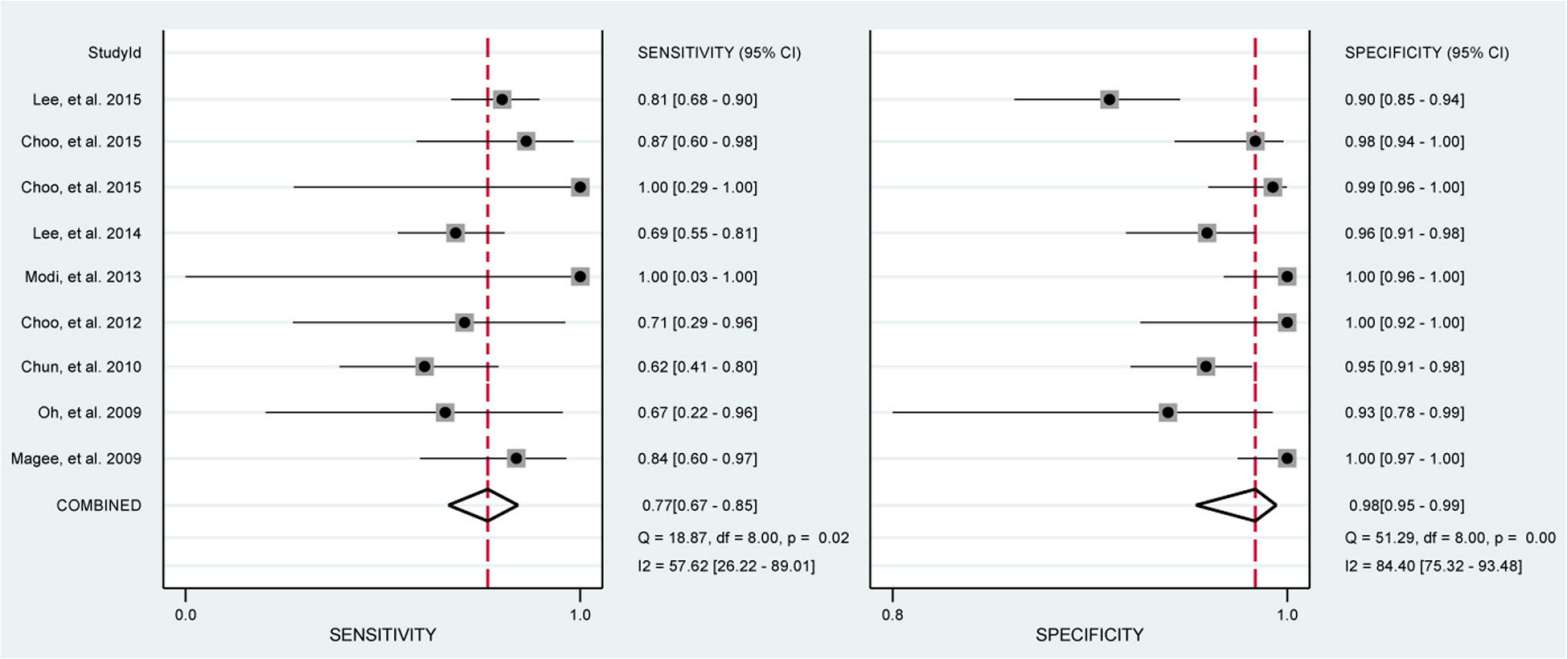
Forest plots of the pooled sensitivity and specificity of MRA to diagnose bursal-sided partial-thickness rotator cuff tears with the corresponding 95% confidence region. Diamonds in the central vertical lines represent pooled sensitivities or specificities with the corresponding 95% confidence interval

**Fig. 3:**
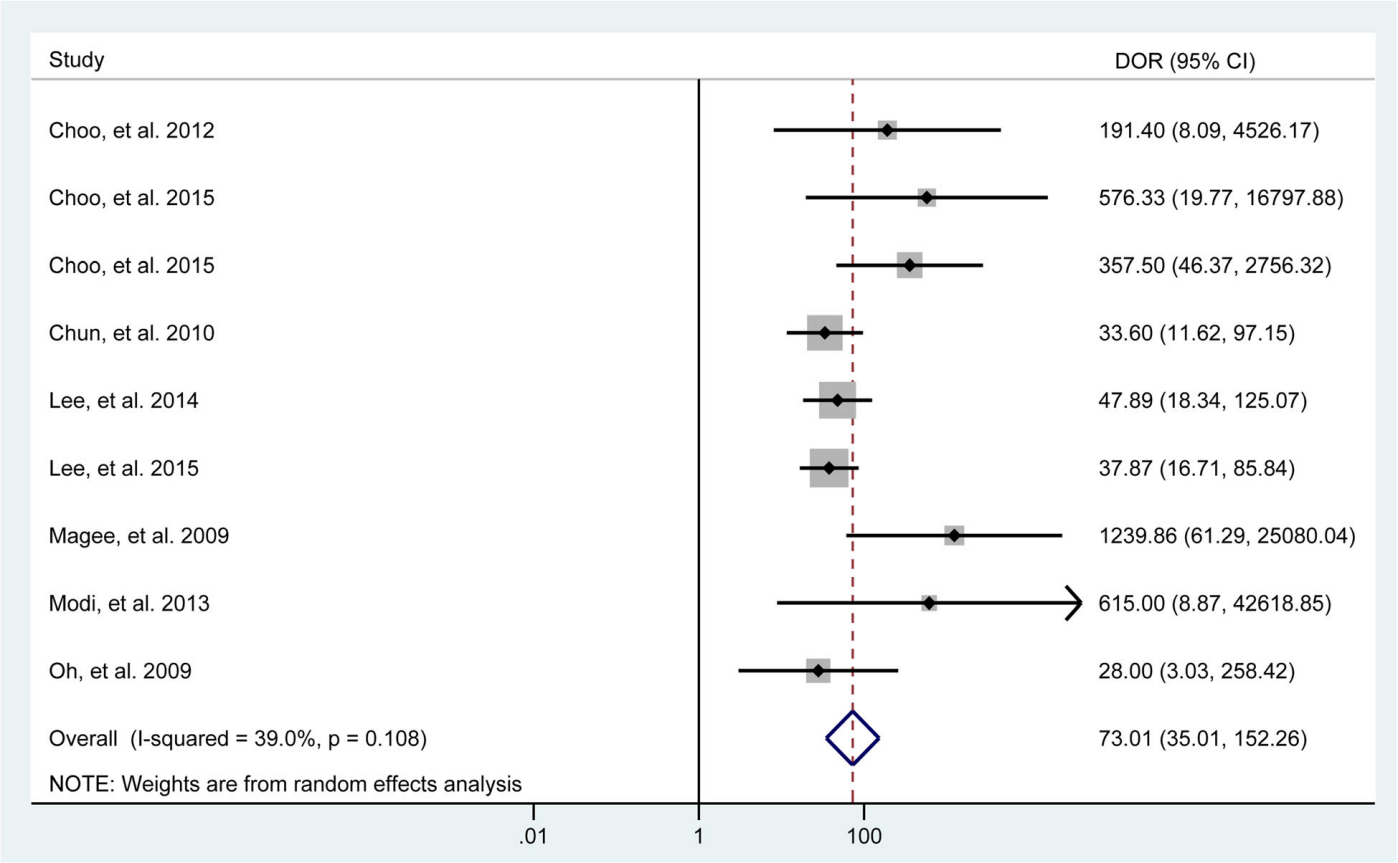
Forest plots of the pooled diagnostic odds ratio (DOR) of MRA to diagnose bursal-sided partial-thickness rotator cuff tears with the corresponding 95% confidence region. Diamonds in the central vertical lines represent pooled DOR with the corresponding 95% confidence interval

**Fig. 4:**
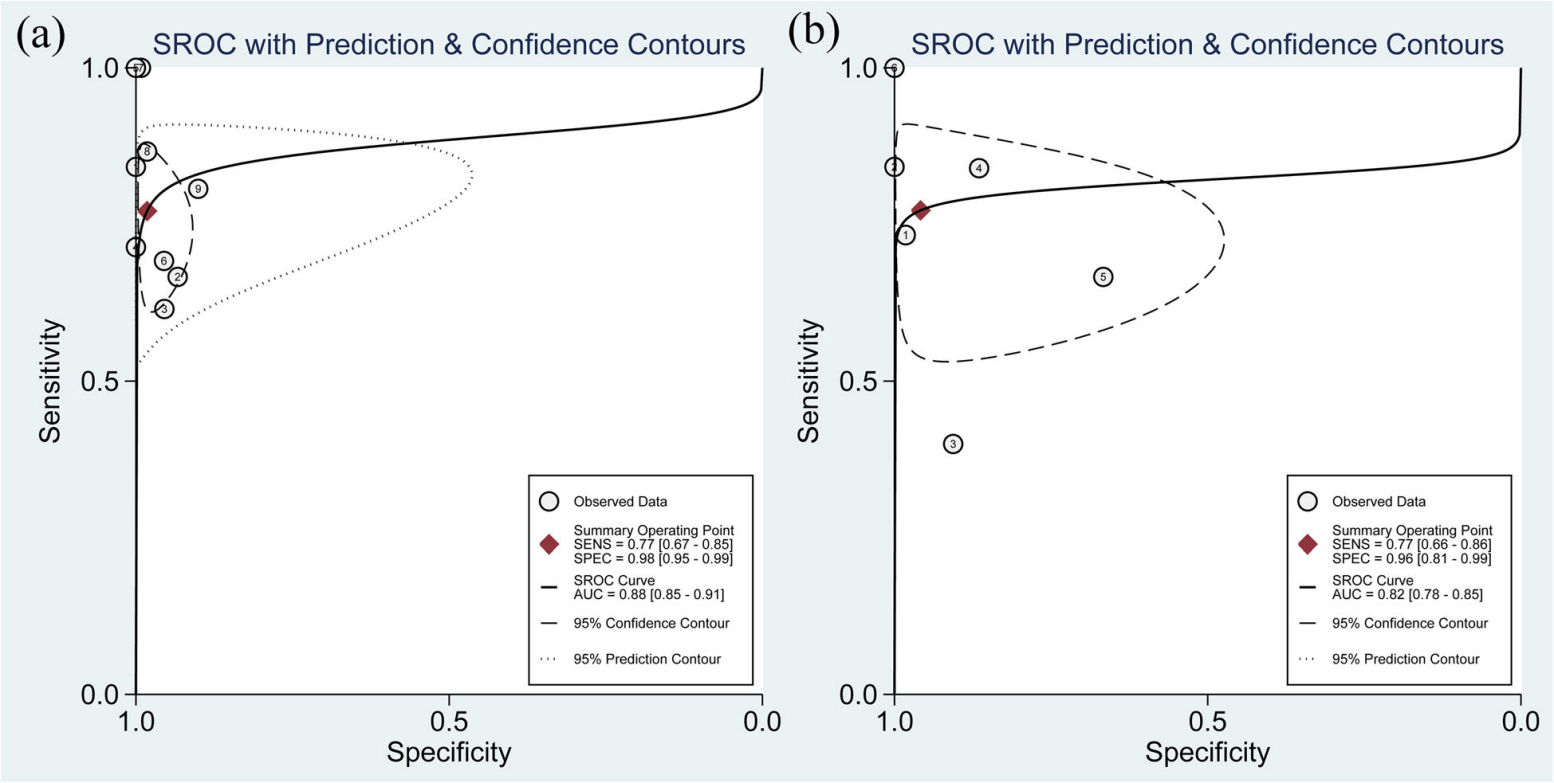
Summarized receiver operating characteristic curve (sROC) of MRA (**a**) and MRI (**b**) to diagnose bursal-sided partial-thickness rotator cuff tears with the corresponding 95% confidence region

**Fig. 5:**
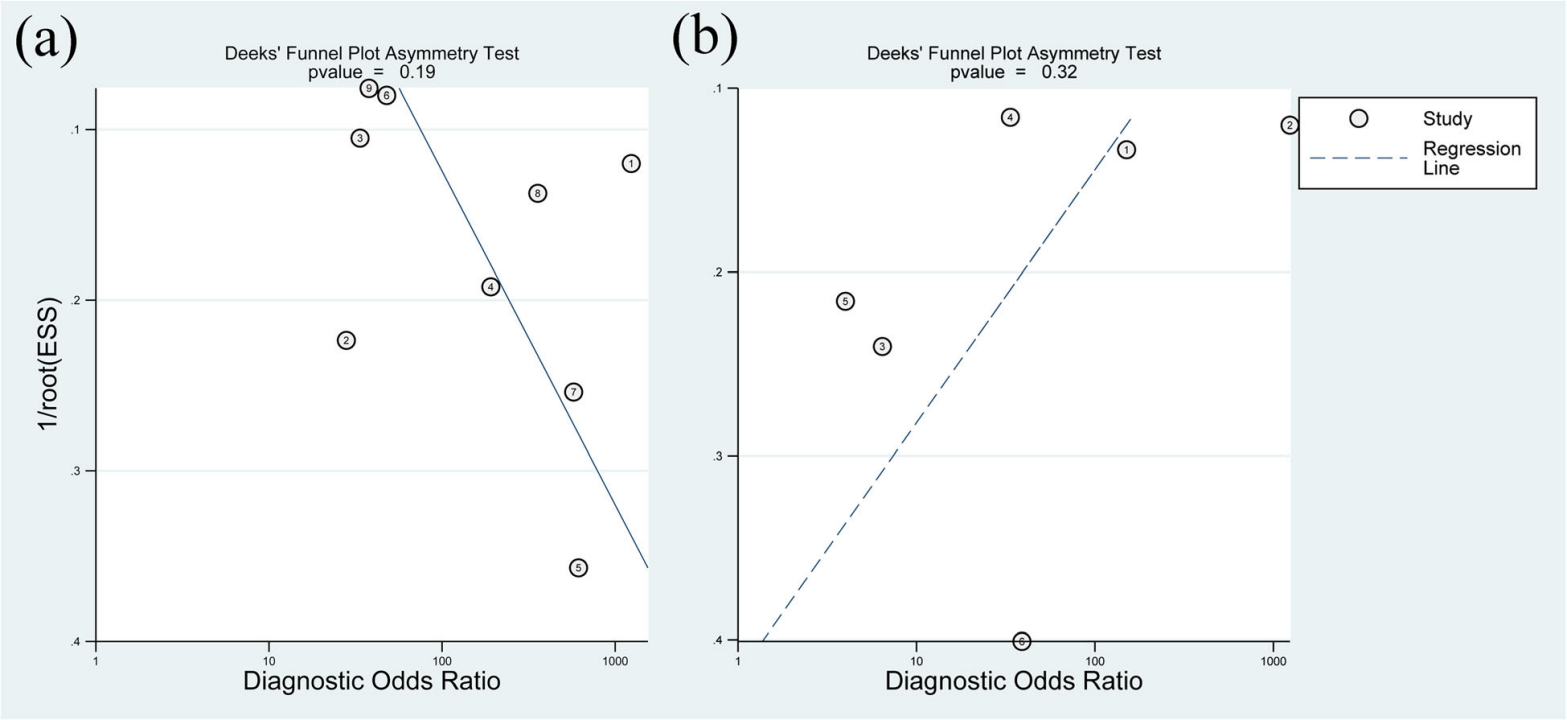
Graphical display of the results of Deek’s test for publication bias of MRA (**a**) and MRI (**b**)

In total, 8 studies [29, 33–38, 40] evaluated the performance of MRA to diagnose bursal-sided partial-thickness RCTs. The pooled sensitivity and specificity were 0.77 (95% CI, 0.67–0.85) and 0.98 (95% CI, 0.95–0.99), respectively (Fig. 2). The pooled PLR, NLR, DOR, and AUC were 43.1 (95% CI, 14.5–128.2), 0.23 (95% CI, 0.16–0.34) (Additional file 1: Figs. S1 and S2), 73.01 (95% CI, 35.01–152.26), and 0.88 (95% CI, 0.85–0.91), respectively (Figs. 3 and 4a). The *I*^2^ statistics for sensitivity and specificity values were 57.62% (95% CI, 26.22–89.01%) and 84.40% (95% CI, 75.32–93.48%), respectively (Fig. 2), indicating substantial heterogeneity among the included studies. Estimation of the Spearman’s correlation coefficient (*p* value = 0.332) indicated the absence of the threshold effect. The results of meta-regression analysis revealed that the number of readers, blinding, and the results of QUADAS-2 score accounted for the heterogeneity of sensitivity and specificity (Additional file 1: Fig. S5), and Deeks’ Funnel Plot Asymmetry Test revealed no publication bias (*p* value = 0.19) (Fig. 5a).

### Diagnostic value of MRI

**Fig. 6:**
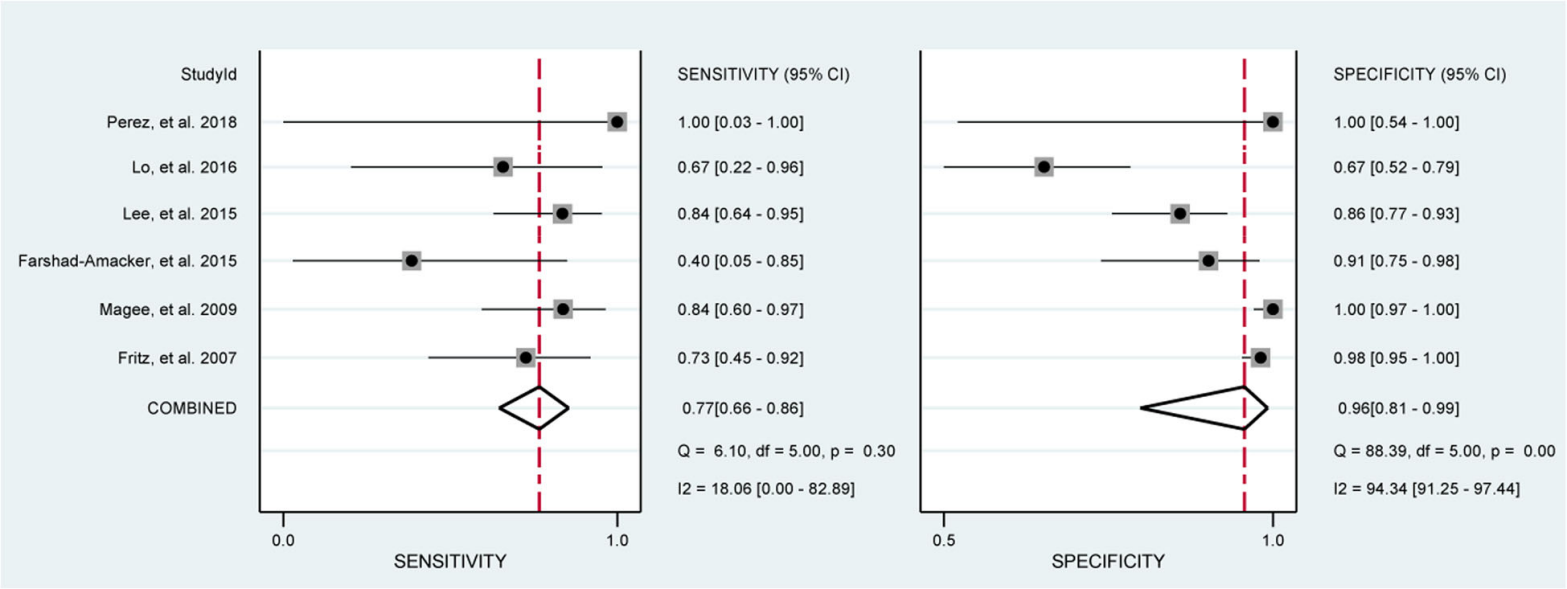
Forest plots of the pooled sensitivity and specificity of MRI to diagnose bursal-sided partial-thickness rotator cuff tears with the corresponding 95% confidence region. Diamonds in the central vertical lines represent pooled sensitivities or specificities with the corresponding 95% confidence interval

**Fig. 7:**
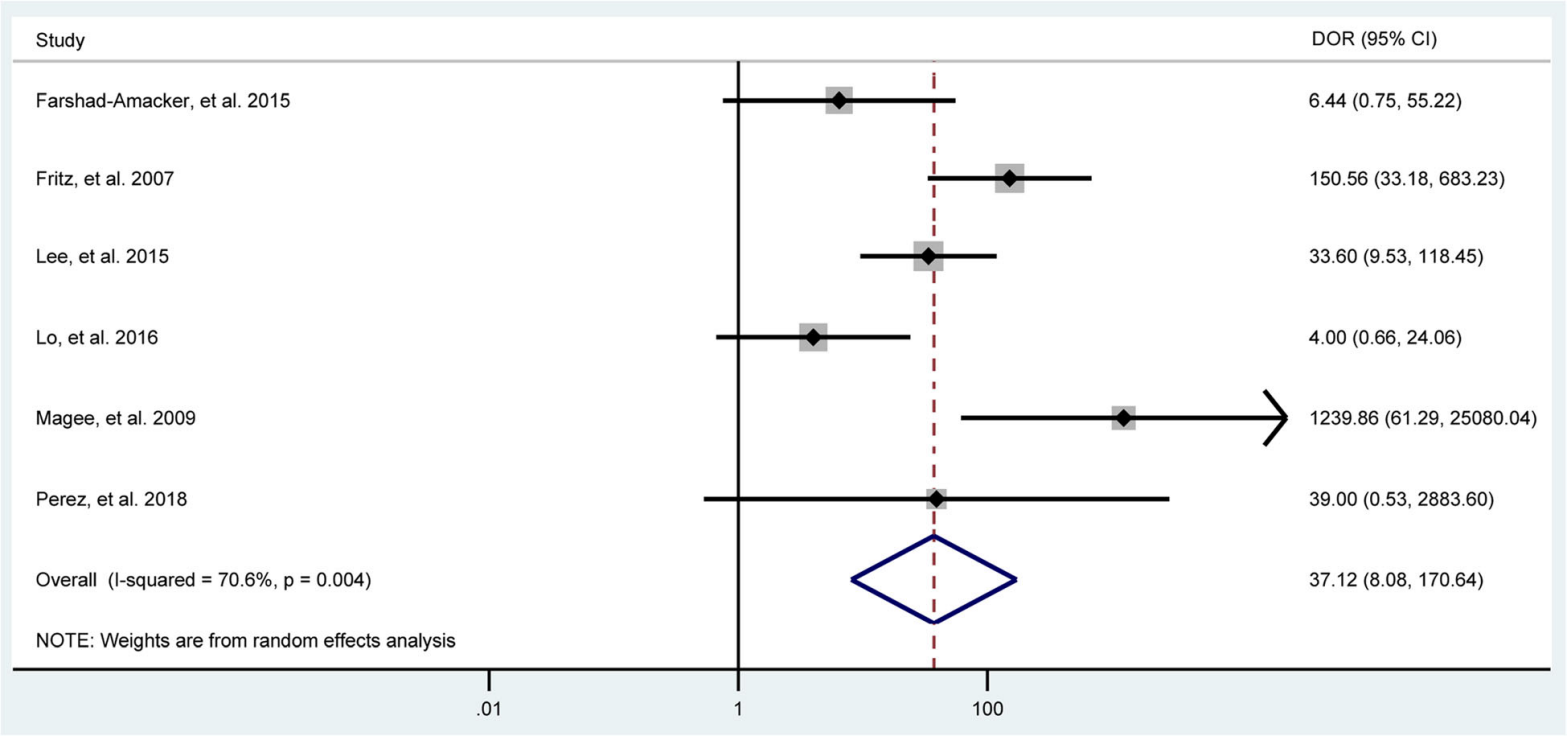
Forest plots of the pooled diagnostic odds ratio (DOR) of MRI to diagnose bursal-sided partial-thickness rotator cuff tears with the corresponding 95% confidence region. Diamonds in the central vertical lines represent pooled DOR with the corresponding 95% confidence interval

In total, 6 studies [32, 33, 39–42] evaluated the performance of MRI to diagnose bursal-sided partial-thickness RCTs. The pooled sensitivity and specificity were 0.77 (95% CI, 0.66–0.86) and 0.96 (95% CI, 0.81–0.99), respectively (Fig. 6). The pooled PLR, NLR, DOR, and AUC were 10.17 (95% CI, 3.00–34.49), 0.31 (95% CI, 0.18–0.54) (Additional file 1: Figs. S3 and S4), 37.12 (95% CI, 8.08–170.64), and 0.82 (95% CI, 0.78–0.85), respectively (Figs. 7 and 4b). The *I*^*2*^ statistics for sensitivity and specificity values were 18.06% (95% CI, 0.01–82.89%) and 94.34% (95% CI, 91.25–97.44%), respectively (Fig. 6), indicating substantial heterogeneity among the included studies. Estimation of the Spearman’s correlation coefficient (*p* value = 0.623) indicated the absence of the threshold effect. The results of meta-regression analysis revealed that publication year and magnetic field strength accounted for the heterogeneity of specificity (Additional file 1: Fig. S6), and the Deeks’ Funnel Plot Asymmetry Test revealed no publication bias (*p* value = 0.32) (Fig. 5b).

## Discussion

Currently, it is still a hot topic that what the best method is to treat partial-thickness rotator cuff tears. The rotator cuff tear completion method leads to faster recovery of function and range of motion but diminished tendon integrity as evidenced by MRI findings [43]. Bursal-sided partial-thickness RCTs have been greatly concerned with the increase of people’s consciousness. Treatment strategy for bursal-sided partial-thickness RCTs depends on both the clinical findings and medical imaging results. The availability and reliability of medical imaging performance, to a great extent, affect the findings under surgery [44]. The surgical treatment of bursal partial-thickness RCTs differs on the basis of whether the intact portion of the tendon is preserved or sacrificed [24]. Multiple studies focused on the assessment of diagnostic value of MRA, MRI, or ultrasound imaging for full- or partial-thickness tears. However, few studies have so far been conducted to rigorously evaluate the diagnostic value of MRA and MRI for the bursal-sided partial-thickness RCTs. Thus, it is essential to evaluate the performance of MRA and MRI for identifying bursal-sided partial-thickness RCTs. Our meta-analysis involving 8 studies for MRA and 6 studies for MRI reveals that MRA and MRI have a similar diagnostic value for identifying bursal-sided partial-thickness RCTs (a sensitivity of 0.77 vs. 0.77; a specificity of 0.98 vs. 0.96; a DOR of 73.09 vs. 37.12; an AUC of 0.88 vs. 0.82).

Compared with interstitial- and articular-sided partial-thickness RCTs, bursal-sided tears can result in more severe shoulder pain [7], possibly because of their association with subacromial impingement [4, 8]. Repeated subacromial impingement of the rotator cuff tendon causes the formation of spurs [8]. One study [5] involving 1029 shoulders revealed that spurs measuring 5 mm or more has a diagnostic value for bursal-side tears. A recent study [45] consisted of 83 patients demonstrated that bursal-sided tears have a higher rate of spurs on the acromion undersurface and impingement sign, which indicated that bursal-sided tears have a highly association with spurs. Oh et al. [46] classified acromions spurs into six types (heel, lateral/anterior traction, lateral/anterior bird beak, and medial) according to the distinct morphology and suggested that the most common heel-type spur might be a risk factor for rotator cuff tears. A distinct finding of the everted type of bursal-sided partial-thickness RCTs on MRI was that the tendon stumps were folded and retracted in the superomedial direction [28]. Kim et al. suggested that the bursal-sided tear is everted and perform MRI to verify if the tear flap is folded.

It has become a hot topic whether or not to inject contrast agents when using MRI for the detection of RCTs. Previously published data have indicated that MRA is more sensitive and specific than MRI or ultrasound imaging for the detection of partial- and full-thickness RCTs [47]. Additionally, MRA has also the most accurate to detecting the labral lesions of shoulder [48, 49]. The reason that MRA has a good diagnostic value in the detection of full-thickness RCTs is because the objective evidence of the leakage of contrast agent is accompanied by a good anatomic resolution and subtle defects depicted by contrast agents [50, 51]. However, MRA, usually with a longer examination time, will bring the infections and adverse complications because of an invasive procedure of the injection of contrast agent [52]. Moreover, the classification and basic properties of the contrast agent will have a huge effect on the diagnostic value of MRA [53]. Numerous studies reported that false positive cases were often triggered by the inflamed tendon [54], and false negative cases were caused by the failure of contrast to pass into the bursa [29, 33]. Therefore, we have a hypothesis that MRA may have a similar diagnostic value for the bursal-sided partial-thickness RCTs. Our results confirmed our hypothesis. Meanwhile, preoperative MRI can provide the surgeon information about the precise location of the tear and the thickness of partial tears, and impending full-thickness tears. The final diagnosis of bursal-sided partial-thickness RCTs was made on a basis of a medical history, physical examination, and reports of medical imaging, which will increase the detection rate. It is a good reason to avoid the potential risk/cost of MRA especially if one suspects a bursal-sided partial-thickness RCTs.

Recently, much more attention has be attracted for the diagnosis and treatments of bursal-sided partial-thickness RCTs because bursal-sided tears can cause more severe shoulder pain that can be surgically treated [4, 7, 8, 29]. Currently, MRI is a noninvasive and reproducible diagnosis method for suspected rotator cuff injuries [55]. Our meta-analysis confirms that MRI has a similar sensitivity and specificity to MRA in the diagnosis of bursal-sided partial-thickness RCTs. It seems that it is not all that beneficial to inject contrast agents when using MRI for the detection of bursal-sided partial-thickness RCTs.

Several limitations exist in this meta-analysis. Evidence of heterogeneity in the data concerning diagnostic accuracy existed among the included studies. The diversity of image planes used for the diagnostic tests and insufficient details regarding various sequences of MRA/MRI and gold standard of analyzing medical imaging reduced the statistical power. Additionally, previous studies concerning rotator cuff tears are mainly comprised of patients suspected of full- and partial-thickness RCTs and not specifically bursal-sided partial-thickness RCTs. Unfortunately, many other imaging diagnostic measures cannot be included in our study because of the limited number of studies, including arthro-computed tomography or ultrasound, which is considered by some surgeons as the gold standard for diagnosing partial-thickness RCTs. Additionally, our results about the diagnostic ability of MRA and MRI should carefully generalize to patients after a post-operation of rotator cuff repair. Another major limitation is that, during data merging, subgroup analyses were not performed based on some important variables such as the sequences of MRA or MRI, imaging planes, the experiences of readers for analyzing medical imaging, slice thickness of MRA/MRI, time from MRI/MRA to gold standard, and the origin of a country. Several minor limitations also merit consideration. First, the number of patients that could be included is small and most of them were retrospective. Second, this study was aimed at diagnosing or excluding bursal-sided partial-thickness RCTs and did not focus on determining other types (articular-sided) of RCTs. Third, whether the results of MRA or MRI were interpreted with blinding to the findings of the reference test was not mentioned in 5 studies, which might influence the interpretation of imaging by medical imaging specialists.

## Conclusion

This meta-analysis reveals that MRA and MRI have similar diagnostic value for the diagnosis of bursal-sided partial-thickness rotator cuff tears.

## Supplementary information

**Additional file 1:** Supplementary Table 1 and Supplementary Figures 1-6.

## Data Availability

All data generated or analyzed during this study are included in this published article.

## Abbreviations

AUC: Area under the receiver operating characteristic curve
CIs: Confidence intervals
DOR: Diagnostic odds ratio
FN: False negative
FP: False positive
MRI: Magnetic resonance imaging
MRA: MR arthrography
NLR: Negative likelihood ratio
PLR: Positive likelihood ratio
PRISMA-DTA: Preferred Reporting Items for a Systematic Review and Meta-analysis of Diagnostic Test Accuracy Studies
QUADAS: Quality assessment of diagnostic accuracy studies
RCTs: Rotator cuff tears
SROC: Summary receiver operating characteristic curve
TN: True negative
TP: True positive
