# Supplementary material for: Diagnostic accuracy of MRA and MRI for the bursal-sided partial-thickness rotator cuff tears: a meta-analysis
Source: J Orthop Surg Res. 2019 Dec 12;14:436. doi: 10.1186/s13018-019-1460-y (PMC6909503; doi:10.1186/s13018-019-1460-y)
Supplement: Supplementary file 1 — Additional file 1: Supplementary Table 1 and Supplementary Figures 1-6. [file 13018_2019_1460_MOESM1_ESM.docx]

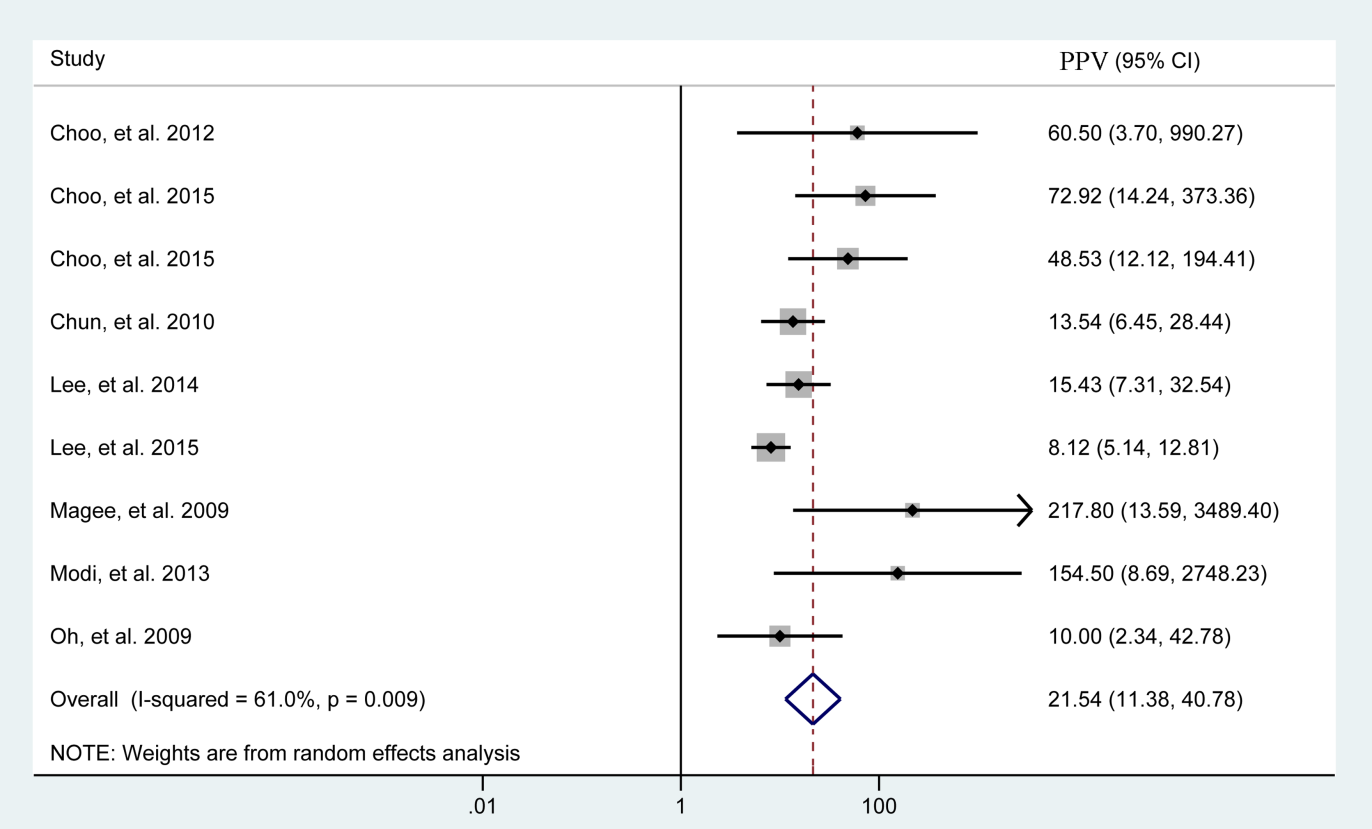


**Supplementary figure 1**. Forest plots of the pooled positive likelihood ratio (PPV) of MRA to diagnose bursal-sided partial-thickness rotator cuff tears with the corresponding 95% confidence region.

Diamonds in the central vertical lines represent pooled positive likelihood ratio with the corresponding 95% confidence interval.


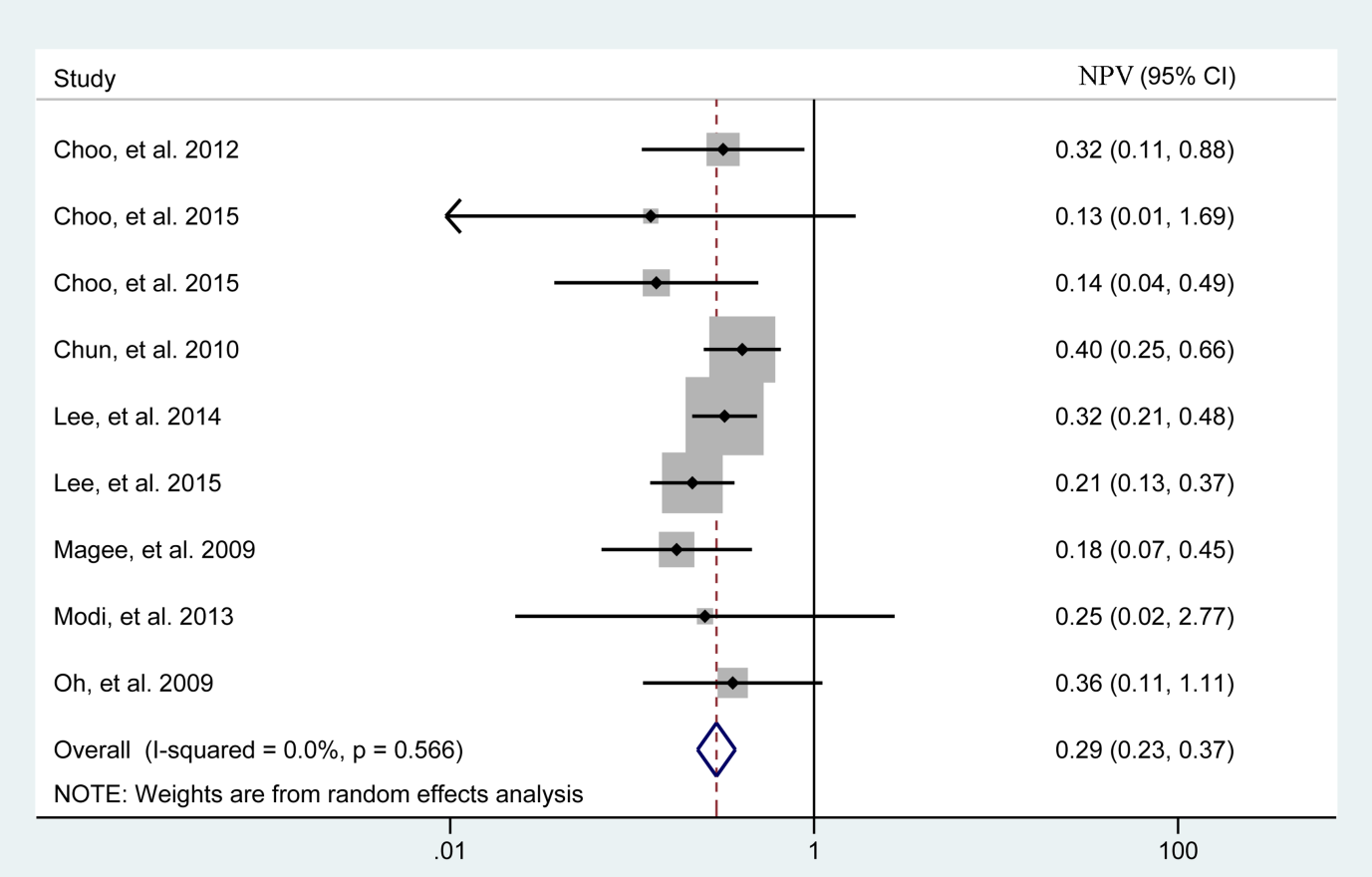


**Supplementary figure 2**. Forest plots of the pooled negative likelihood ratio (NPV) of MRA to diagnose bursal-sided partial-thickness rotator cuff tears with the corresponding 95% confidence region.

Diamonds in the central vertical lines represent pooled negative likelihood ratio with the corresponding 95% confidence interval.


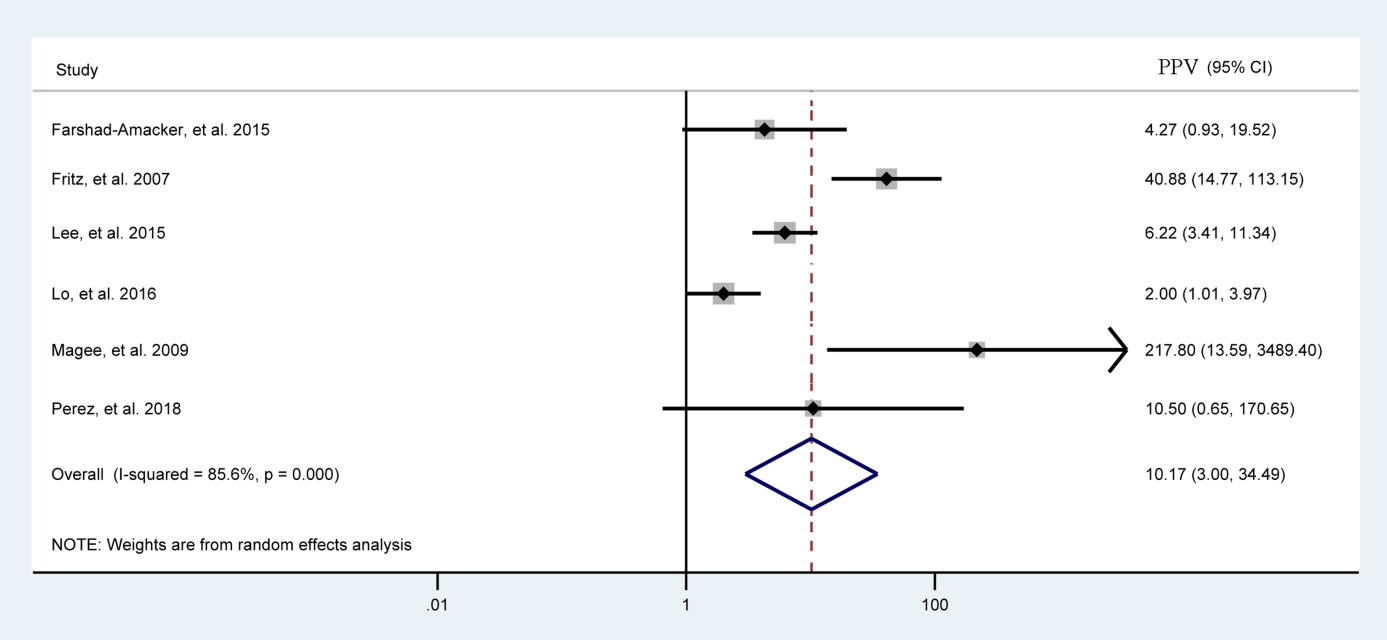


**Supplementary figure 3**. Forest plots of the pooled positive likelihood ratio (PPV) of MRI to diagnose bursal-sided partial-thickness rotator cuff tears with the corresponding 95% confidence region.

Diamonds in the central vertical lines represent pooled positive likelihood ratio with the corresponding 95% confidence interval.


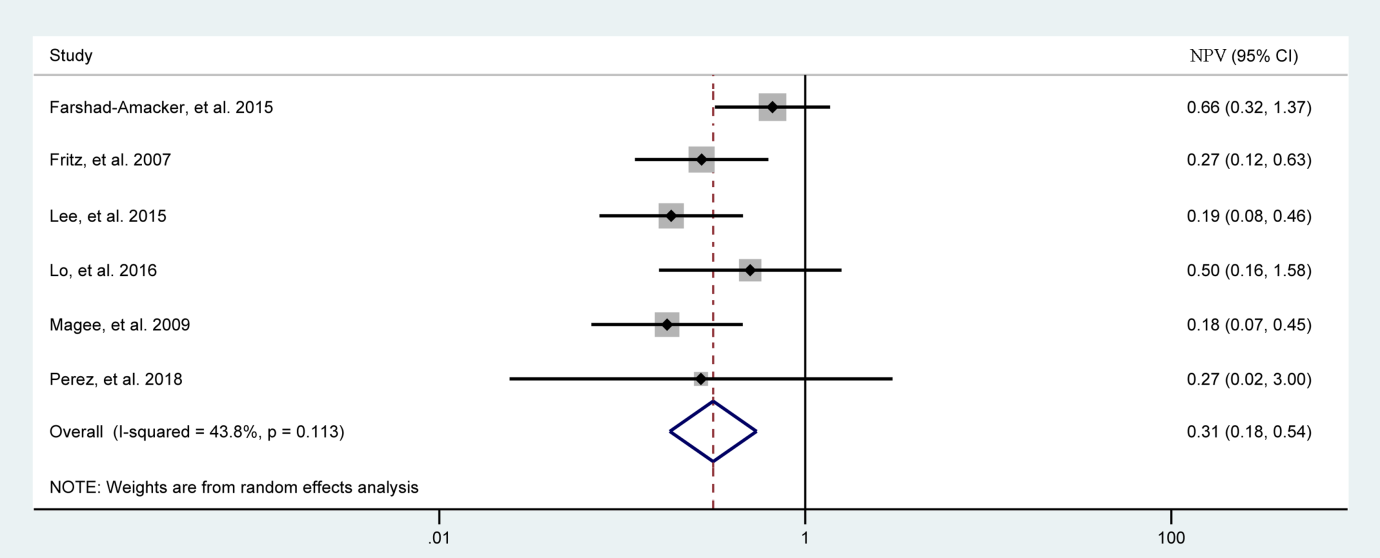


**Supplementary figure 4**. Forest plots of the pooled negative likelihood ratio (NPV) of MRI to diagnose bursal-sided partial-thickness rotator cuff tears with the corresponding 95% confidence region.

Diamonds in the central vertical lines represent pooled negative likelihood ratio with the corresponding 95% confidence interval.


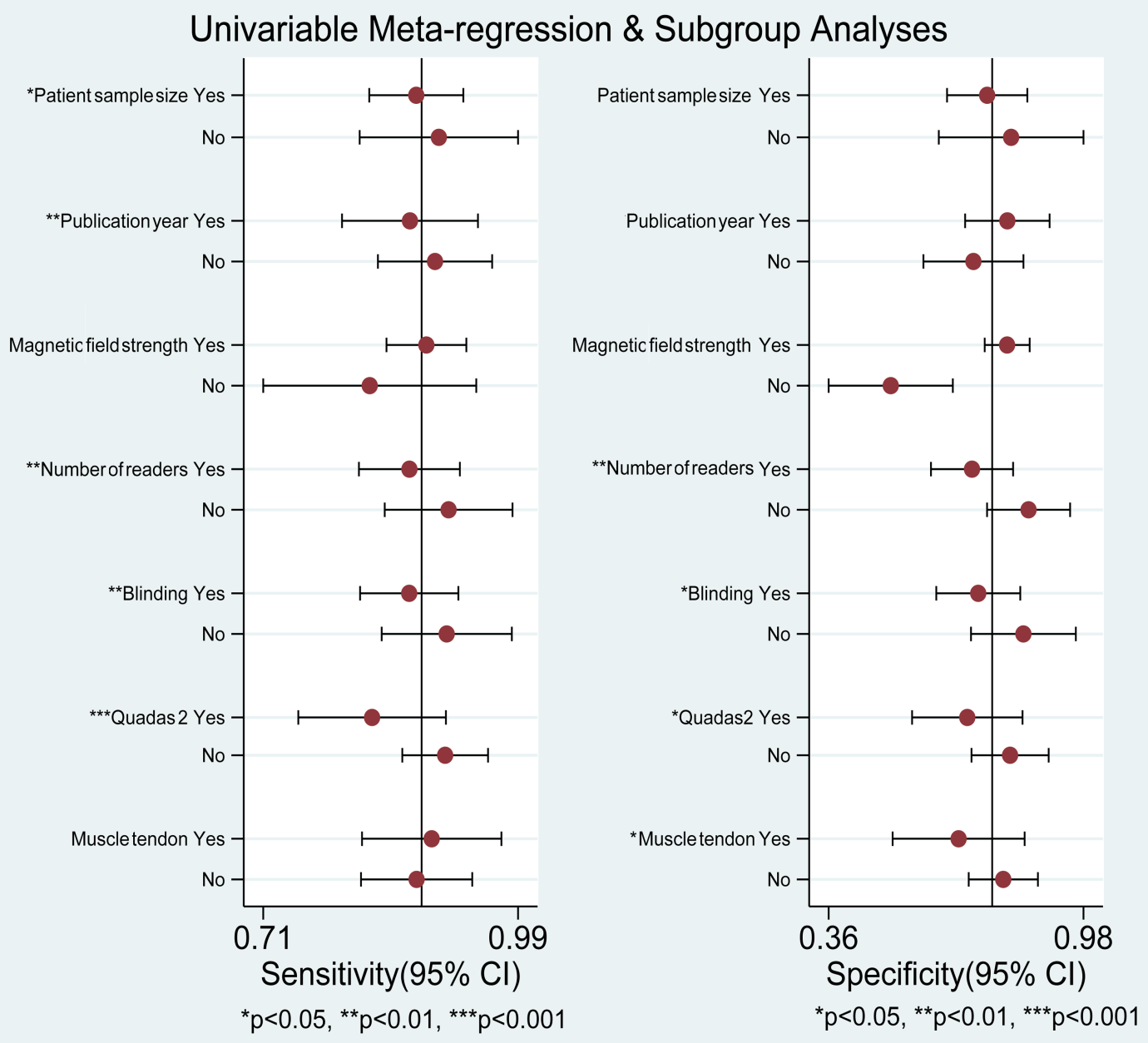


**Supplementary figure 5**. Graphical display of the results of univariable meta-regressions of MRA.


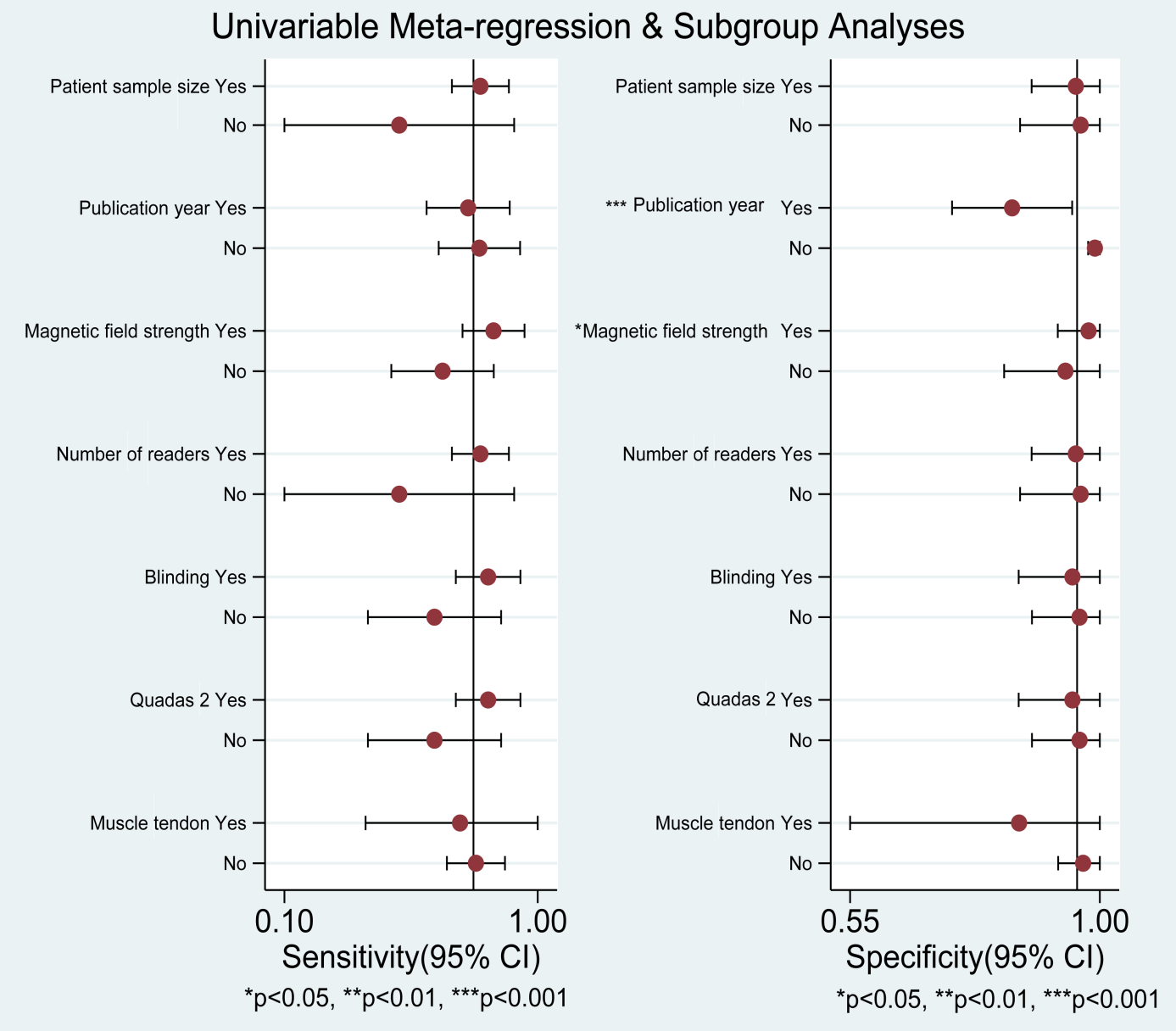


**Supplementary figure 6**. Graphical display of the results of univariable meta-regressions of MRI.

| **Supplementary table 1. The accuracy of MRA and MRI for detection of bursal partial-thickness rotator cuff tears.** | | | | | | | | |
| --- | --- | --- | --- | --- | --- | --- | --- | --- |
| Study | Method | Magnetic field strength | Sensitivity (95% CI) | Specificity  (95% CI) | PLR  (95% CI) | NLR (95% CI) | DOR (95% CI) | AUC (95% CI) |
| Magee, et al. 2009 | MRA | 3.0 T | 0.84  (0.60, 0.97) | 1.00  (0.97, 1.00) | 217.80  (13.59, 3489.40) | 0.18  (0.07, 0.45) | 1239.86  (61.29, 25080.0) | - |
| Oh, et al. 2009 | MRA | 3.0 T | 0.67  (0.22, 0.96) | 0.93  (0.78, 0.99) | 10.00  (2.34, 42.78) | 0.36  (0.11, 1.11) | 28.00  (3.03, 258.42) | - |
| Chun, et al. 2010 | MRA | 1.5 T | 0.62  (0.41, 0.80) | 0.95  (0.91, 0.98) | 13.54  (6.45, 28.44) | 0.40  (0.25, 0.66) | 33.60  (11.62, 97.15) | - |
| Choo, et al. 2012 | MRA | 3.0 T | 0.71  (0.29, 0.96) | 1.00  (0.92, 1.00) | 60.50  (3.70, 990.27) | 0.32  (0.11, 0.88) | 191.40  (8.09, 4526.17) | - |
| Modi, et al. 2013 | MRA | 3.0 T | 1.00  (0.03, 1.00) | 1.00  (0.96, 1.00) | 154.50  (8.69, 2748.23) | 0.25  (0.02, 2.77) | 615.00  (8.87, 42618.85) | - |
| Lee, et al. 2014 | MRA | 3.0 T | 0.69  (0.55, 0.81) | 0.96  (0.91, 0.98) | 15.43  (7.31, 32.54) | 0.32  (0.21, 0.48) | 47.89  (18.34, 125.07) | - |
| Choo, et al. 2015 | MRA | 3.0 T | 1.00  (0.29, 1.00) | 0.99  (0.96, 1.00) | 72.92  (14.24, 373.36) | 0.13  (0.01, 1.69) | 576.33  (19.77, 16797.8) | - |
| Choo, et al. 2015 | MRA | 3.0 T | 0.87  (0.60, 0.98) | 0.98  (0.94, 1.00) | 48.53  (12.12, 194.41) | 0.14  (0.04, 0.49) | 357.50  (46.37, 2756.32) | - |
| Lee, et al. 2015 | MRA | 3.0 T | 0.81  (0.68, 0.90) | 0.90  (0.85, 0.94) | 8.12  (5.14, 12.81) | 0.21  (0.13, 0.37) | 37.87  (16.71, 85.84) | - |
| Pooled estimate |  |  | **0.77**  **(0.67, 0.85)** | **0.98**  **(0.95, 0.99)** | **43.1**  **(14.5, 128.2)** | **0.23**  **(0.16, 0.34)** | **73.01**  **(35.01, 152.26)** | **0.88**  **(0.85, 0.91)** |
| Fritz, et al. 2007 | MRI | 1.5 T | 0.73  (0.45, 0.92) | 0.98  (0.95, 1.00) | 40.88  (14.77, 113.15) | 0.27  (0.12, 0.63) | 150.56  (33.18, 683.23) | - |
| Magee, et al. 2009 | MRI | 3.0 T | 0.84  (0.60, 0.97) | 1.00  (0.97, 1.00) | 217.80  (13.59, 3489.4) | 0.18  (0.07, 0.45) | 1239.9  (61.29, 25080.3) | - |
| Farshad-Amacker, et al. 2015 | MRI | 1.5/3.0 T | 0.40  (0.05, 0.85) | 0.91  (0.75, 0.98) | 4.27  (0.93, 19.52) | 0.66  (0.32, 1.37) | 6.44  (0.75, 55.22) | - |
| Lee, et al. 2015 | MRI | 3.0 T | 0.84  (0.64, 0.95) | 0.86  (0.77, 0.93) | 6.22  (3.41, 11.34) | 0.19  (0.08, 0.46) | 33.60  (9.53, 118.45) | - |
| Lo, et al. 2016 | MRI | 1.5 T | 0.67  (0.22, 0.96) | 0.67  (0.52, 0.79) | 2.00  (1.01, 3.97) | 0.50  (0.16, 1.58) | 4.00  (0.67, 24.07) | - |
| Perez, et al. 2018 | MRI | 1.5 T | 1.00  (0.03, 1.00) | 1.00  (0.54, 1.00) | 10.50  (0.65, 170.65) | 0.27  (0.02, 3.00) | 39.00  (0.53, 2883.6) | - |
| Pooled estimate |  |  | **0.77**  **(0.66, 0.86)** | **0.96**  **(0.81, 0.99)** | **10.17**  **(3.00, 34.49)** | **0.31**  **(0.18, 0.54)** | **37.12**  **(8.08, 170.64)** | **0.82**  **(0.78, 0.85)** |
| MRA magnetic resonance angiography, MRI magnetic resonance imaging, T tesla, PLR positive likelihood ratio, NLR negative likelihood ratio, DOR diagnostic odd ratio, AUC area under curve, CI confidence interval. | | | | | | | | |
